# First Line Therapy in 2014

**DOI:** 10.1186/1471-2334-14-S2-S3

**Published:** 2014-05-23

**Authors:** Mark Nelson

**Affiliations:** 1 Chelsea and Westminster Hospital, Department of HIV and Genitourinary Medicine, London, UK

## 

The choice of antiretroviral therapy for those naïve to therapy is vast, with over 30 drugs available for the treatment of HIV disease. Although international and national guidelines often give recommended preferred choices for commencement of therapy, it is important that treatment is always individualised. Therefore for the individual patient, it may be necessary to utilise treatments outside of the guidelines which too often refer to the near perfect patient.

Choice of antiretroviral therapy must be based on several factors. These would include comparative trials on efficacy. However the toxicity of the agents both singly and in combination may be the most important in individualisation of therapy, due to patient wishes and associated co-morbidities. The ability for the patient to comply with therapy may be made easier with once daily therapy although the role of single tablet regimens remains controversial. Together efficacy, toxicity and adherence contribute to the potency of the antiretroviral regimen, where potency is defined as the ability to succeed.

Other factors have become increasingly important. There are increasing reports of primary resistance in several European areas, which clearly may impact on the choice of therapy, particularly within the non-nucleoside class. In addition, with an ageing HIV population including those newly diagnosed, potential drug interactions will become an increasingly important factor. In several countries the issue of cost has now become an essential driver towards choice of therapy, particularly with the advent of generic drugs.

The majority of individuals will receive two nucleoside reverse transcriptase inhibitors in combination with either a ritonavir boosted protease inhibitor, a non-nucleoside reverse transcriptase inhibitor most commonly efavirenz, or an integrase inhibitor.

A recent large scale study performed by the ACTG, suggested that overall success may be higher with an integrase inhibitor, when compared with a ritonavir boosted protease inhibitor, but closer examination of these results shows that this was mostly driven by toxicities associated with the protease inhibitors and a switch from these drugs was not associated with a lower rate of long-term success.

Several studies have examined novel treatments include nucleoside sparing regimens. A recent study from the NEAT Network suggested that a combination of raltegravir, ritonavir and darunavir performed as well as a combination of truvada, ritonavir and darunavir. Again closer inspection of these results would suggest that the nucleoside sparing regimen did not perform as well when stressed both at high viral load and low CD4 count.

The initial choice of antiretroviral therapy is perhaps one of the most important choices for the patient to make. This should never be rushed, should be performed within a partnership of patient and physician, and should always reflect the needs of the individual patient not the prescribing physician.

